# ReadXplorer—visualization and analysis of mapped sequences

**DOI:** 10.1093/bioinformatics/btu205

**Published:** 2014-04-30

**Authors:** Rolf Hilker, Kai Bernd Stadermann, Daniel Doppmeier, Jörn Kalinowski, Jens Stoye, Jasmin Straube, Jörn Winnebald, Alexander Goesmann

**Affiliations:** ^1^Institute of Medical Microbiology, Justus-Liebig-University, 35392 Giessen, Germany, ^2^Faculty of Biology, ^3^Institute for Bioinformatics, Center for Biotechnology, ^4^Computational Genomics, Center for Biotechnology, ^5^Technology Platform Genomics, Center for Biotechnology, ^6^Genome Informatics, Faculty of Technology, Bielefeld University, 33615 Bielefeld, Germany and ^7^Bioinformatics and Systems Biology, Faculty of Biology and Chemistry, Justus-Liebig-University, 35392 Giessen, Germany

## Abstract

**Motivation: **Fast algorithms and well-arranged visualizations are required for the comprehensive analysis of the ever-growing size of genomic and transcriptomic next-generation sequencing data.

**Results:** ReadXplorer is a software offering straightforward visualization and extensive analysis functions for genomic and transcriptomic DNA sequences mapped on a reference. A unique specialty of ReadXplorer is the quality classification of the read mappings. It is incorporated in all analysis functions and displayed in ReadXplorer's various synchronized data viewers for (i) the reference sequence, its base coverage as (ii) normalizable plot and (iii) histogram, (iv) read alignments and (v) read pairs. ReadXplorer's analysis capability covers RNA secondary structure prediction, single nucleotide polymorphism and deletion–insertion polymorphism detection, genomic feature and general coverage analysis. Especially for RNA-Seq data, it offers differential gene expression analysis, transcription start site and operon detection as well as RPKM value and read count calculations. Furthermore, ReadXplorer can combine or superimpose coverage of different datasets.

**Availability and implementation: **ReadXplorer is available as open-source software at http://www.readxplorer.org along with a detailed manual.

**Contact:** rhilker@mikrobio.med.uni-giessen.de

**Supplementary information: **Supplementary data are available at *Bioinformatics* online.

## 1 INTRODUCTION

Next-generation sequencing (NGS) technologies (Niedringhaus *et al.*, 2011) like the well-established and continuously improved sequencing by synthesis (Bennett, 2004) and pyrosequencing (Margulies *et al.*, 2005) or third-generation sequencing technologies like SMRT (Eid *et al.*, 2009) and semiconductor sequencing (Rothberg *et al.*, 2011) generate an ever-growing amount of genomic and transcriptomic sequence data. Currently, single deep sequencing datasets even for prokaryotic organisms can consist of tens of millions of read sequences. At the same time, sequencing costs have fallen to >1000 dollars for a complete human genome.

Analyzing differences and commonalities (Blom *et al.*, 2009) or validating and refining the region annotations (Schrimpe-Rutledge *et al.*, 2012) of genomes is a task that has become routine in virtually all research institutes in the fields of microbiology and biotechnology because of the affordable costs of such an analysis (Liu *et al.*, 2012). The same holds for massive parallel complementary DNA (cDNA) sequencing (RNA-Seq) experiments, which isolate a snapshot of the transcriptome of an organism (Wang *et al.*, 2009) under prevailing or induced environmental conditions. These RNA-Seq datasets can be studied with respect to the abundance of expressed genes and non-coding RNAs under different environmental conditions. Besides differential gene expression analysis, the identification of transcription start sites (TSSs) can be improved by using 5′-enriched RNA-Seq datasets (Sharma *et al.*, 2010), which contain only the first bases of the sequenced transcripts.

Without automated analyses, advanced visualization and efficient handling of large datasets, it would be a tedious and interminable task to investigate these data. Hence, efficient and scalable algorithms are needed for the automated analysis and evaluation. To quickly browse the tremendous amounts of data and facilitate drawing conclusions, visualization of DNA sequence datasets has to be easy to use. Analysis results need to be visualized immediately, and important regions of a genome or transcriptome have to be accessible instantly.

The list of popular published applications already available for visualizing short read mapping data in a genomic context includes SAVANT (Fiume *et al.*, 2010), GenomeView (Abeel *et al.*, 2012), IGV (Thorvaldsdóttir *et al.*, 2013), IGB (Nicol *et al.*, 2009) and Artemis (Carver *et al.*, 2012). All of these tools are desktop genome viewers, which are mainly designed for interactively displaying and handling various NGS data formats.

Artemis was first a genome annotation tool, which has been extended to visualize high-throughput read mapping data, variant calls and user-defined input types. IGV is a flexible viewer for various NGS data formats but does not include automatic analysis capabilities. IGB is a viewer for real-time zooming and panning through genomes. It can display annotations and position-specific numerical graphs, e.g. calculated from coverage data. GenomeView is a dynamic short read and whole genome alignment browser with capabilities for editing reference features. SAVANT is a genome viewer capable of visualizing different NGS data formats and includes plug-ins for analysis functions. Among these viewers, SAVANT and GenomeView are extensible through plug-in interfaces. Comprehensive analysis functions for short read mapping data are only included in SAVANT.

Here, plug-ins were developed, for example, for single nucleotide polymorphism (SNP) detection and RNA-Seq analyses. There are several more useful published and also unpublished tools, like SeqMonk (http://www.bioinformatics.babraham.ac.uk/projects/seqmonk/), available. Many of the published tools are listed in (Abeel *et al.*, 2012).

All currently available applications have in common that they display read mapping data as it is stored in read mapping files. These files, however, provide no information about the quantity and quality of each single read mapping. Such a classification of mapped reads, however, is a crucial step toward increasing the reliability of mapping analyses, as reads can map multiple times on the reference either with the same or with different amounts of mismatches.

To add this knowledge and allow the user to freely decide upon the read type to be used for an analysis, we developed our software ReadXplorer. It is an interactive genome browser with broad analytic capabilities built around this read mapping classification. Moreover, it contains novel analysis methods and visualization modes for NGS datasets.

## 2 IMPLEMENTATION

ReadXplorer is implemented in Java 1.7 and based on the Netbeans rich client platform (http://netbeans.org/features/platform). Therefore, it is a desktop client application running on all major operating systems such as Windows, Linux or Mac OS.

In this chapter, we describe the database and stream-driven data model, the main software architecture, the modular user interface, various visualizations and the analysis capabilities of ReadXplorer for large NGS datasets.

### 2.1 Software architecture and tiers

ReadXplorer is implemented as a rich client application using a modular programming structure, which allows for simple integration of new plug-ins and modules.

The core features of ReadXplorer are bundled in the form of individual Netbeans modules (NBMs) in a module suite. All additional functionalities, such as analysis functions, are contained in NBMs in an add-on module suite. These add-ons are embedded into ReadXplorer as plug-ins via an integrated plug-in manager. This allows for easy extension of our rich client application: new NBMs developed by external programmers only have to be placed into ReadXplorer's update folder. During the next start of the software, they are immediately integrated in the current ReadXplorer version. This behavior guarantees easy integration of new plug-ins, which fits highly specialized user needs.

**Fig. 1.**
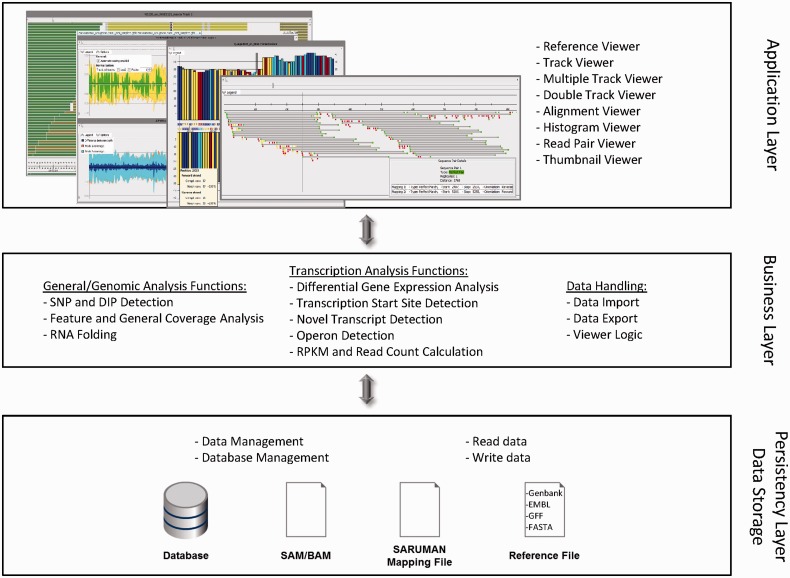
ReadXplorer tiers. This figure displays the global three-tier architecture of ReadXplorer. The application tier is responsible for data visualization and user input, whereas the business tier contains all logic associated with analysis functions and data import and export. The NGS data are maintained, written and read by the persistency tier. It either interacts with a database, a file or both

The software is composed of a classical three-tier architecture, of which a high-level overview is given in Figure 1.

We provide a well-defined application programming interface featuring classes for the development of specialized data viewers. Functionality comprises, for example, the synchronization of data between different data viewers.

Requests for any kind of data are handled by the business tier, whereas the persistency tier is subsequently responsible for executing necessary data queries. When a query succeeds, the persistency tier passes the result in generic data containers to the business tier. This behavior guarantees independency and exchangeability of each tier and its components.

### 2.2 Data model

A ReadXplorer project is always based on either an H2 (www.h2database.com) or MySQL (www.mysql.com) database. We recommend using ReadXplorer with H2 because it is file based, thus guaranteeing flexibility for the users. An H2 database can be copied and opened anywhere where the ReadXplorer software is available, without the necessity of running a database server.

ReadXplorer currently supports the import of a reference sequence in five different formats: Genbank, EMBL, GFF2, GFF3 and plain FASTA. In any case, after import, all reference features like genes, coding sequences and different RNA types are stored in the database.

The read mapping data can be created by any mapping tool generating SAM or BAM files (Li *et al.*, 2009), like bwa (Li and Durbin, 2009), or with SARUMAN (Blom *et al.*, 2011), generating JOK output files. Afterward, the read mapping data can be imported into ReadXplorer and are classified according to the following two sections: Sections 2.2.1 and 2.2.2. For reliable analyses, the classification is a crucial step because sequencing reads often map to more than one region of the reference sequence with different amounts of mismatches, for instance, in repetitive regions. Therefore, not all mapped reads are equally trustworthy for each analysis, but these reads might still be useful for other analyses. During import, the classification data are stored in a BAM file, which is an extended copy of the original file. Subsequently, ReadXplorer only works on these extended files. Within ReadXplorer, an imported mapping dataset is called *track*. The contents of a database are organized in and quickly accessible via a dashboard (Supplementary Fig. S1B).

#### 2.2.1 Read mapping classification

**Fig. 2.**
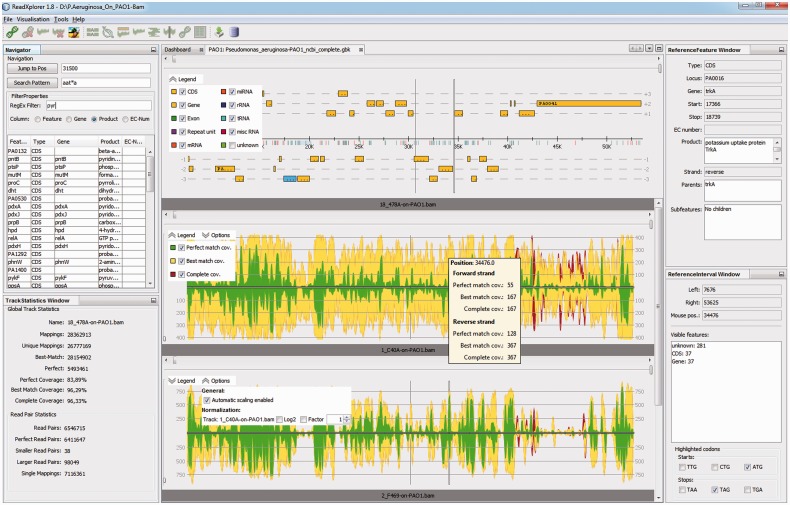
ReadXplorer main GUI. This figure displays the main components of ReadXplorer. Reference and track panels are placed in the center. Their legend enables the user to select the displayed data. Besides that, a track viewer contains automatic scaling and normalization options. The coverage is color coded according to its mapping class: Perfect (green), Best Match (yellow) and Common mappings (red). In the left top corner, the navigator panel allows quick pattern searching, feature filtering and browsing the reference. Below the navigator, the global statistics of the currently selected track are shown. On the right hand side, the reference feature panel displays the details of the currently selected reference feature, and the reference interval panel shows a summary of the genomic features in the currently viewed reference interval. Furthermore, the reference interval panel allows highlighting start and stop codons in the reference according to a selectable NCBI genetic standard code (http://www.ncbi.nlm.nih.gov/Taxonomy/Utils/wprintgc.cgi)

Read mapping data have to be provided in SAM, BAM (Li *et al.*, 2009) or SARUMAN (Blom *et al.*, 2011) output format. In contrast to all other available programs, ReadXplorer classifies the read mapping data during the import process by mapping quality measures and read pair concordance. A read mapped to a certain reference position is called *mapping* in the following. For each read, the number of mappings on the reference is counted along with the lowest number of mismatches found among the various mappings of that read. Uniquely mapped reads (called *unique mappings*) or reads with a certain amount of mismatches can thus easily be queried. The mappings are further classified into three classes: all mappings without any mismatches belong to the *Perfect Match* class. A mapping that cannot be placed to another position in the reference with fewer mismatches than at the current position, belongs to the *Best Match* class. All remaining mappings belong to the *Common Match* class. Beginning with the Perfect Match class, these classes are proper subsets of the next larger class. Note that the same read can map to different reference positions with the same number of mismatches. Therefore, also the Perfect and Best Match classes do not necessarily contain reads that are uniquely mapped to one position of the reference. As mentioned earlier, these mappings can easily be accessed by the mapping count stored in each mapping. Besides that, we define *Single Best Match* mappings as multiple mapped reads with a unique best match position in the reference sequence. To gain access to the Single Best Match mappings, we store a flag in each mapping. This classification approach is unique and an advantage of ReadXplorer. By showing the mapping classes in the graphical user interface (GUI; see Fig. 2 and Section 2.3), we allow the user to select for each analysis which mapping classes are important and should be included in the analysis. Additionally, our unique classification approach allows incorporation of mappings in analyses, which would be excluded by other programs.

#### 2.2.2 Read pair classification

For paired-end or mate pair mappings, a read pair classification algorithm was developed, which takes into account all occurrences of each read. In the following, we distinguish between all mappings of the two reads of a pair, which we call *mapping pair*, and two mappings of a mapping pair classified as *read pair*. In general, the algorithm classifies the mappings again into three different classes: *Perfect* read pairs with correct orientation and a pair distance within a certain range defined by the user, *Distorted* read pairs, whose distance deviates from the perfect distance interval and/or whose orientation is incorrect and *Single Mappings*, whose partner could not be mapped on the reference.

A special case of Single Mappings occurs when one or both reads of a mapping pair also map to other regions of the reference, but they cannot be associated with a Perfect or Distorted read pair. There might be more than one Perfect read pair for the same mapping pair. Further, the mapping classes Perfect, Best Match and Common are considered in the read pair identification algorithm to improve the accuracy of correct predictions. For each mapping pair, Perfect mapping read pairs are preferred to Best Match, and both are preferred to Common mapping read pairs. The read pairs are visualized in a special Read Pair Viewer (see Section 2.3 and Supplementary Fig. S2A). Table 1 gives an overview of the classification properties.

**Table 1.** List of possible classifications of all mappings of the two reads that belong to a mapping pair according to their respective mapping count on a reference sequence

Number of mappings	Read pair classification
1	Single Mapping
2	Pair
>2, including at least one Perfect pair	Perfect pairs are stored, remaining mappings are stored as Single Mappings
>2, including at least one smaller distance pair; may also contain Perfect pairs	Perfect pairs are stored, largest smaller distance pair for each region is stored, remaining mappings are stored as Single Mappings
>2, including only larger distance mappings	All mappings stored as Single Mappings

*Note*: The first column shows the cumulative mapping count of both reads and additional pairing properties. The second column depicts the classification of the mappings in the classes *read pair* or *Single Mapping*. The affiliation of each mapping to its mapping pair is always preserved to enable retrieval of all mappings of a mapping pair.

### 2.3 Interface and visualization

ReadXplorer's ease of use is facilitated by a modular docking framework for all GUI components, which is based on the Netbeans integrated development environment. All panels can always be maximized, minimized and restructured in various ways, enabling the user to perform simultaneous comparisons of different datasets or browse different genomic regions of the same dataset. Furthermore, the last user-defined layout of the visual components is always restored after restarting the software.

The novel mapping classification introduced in Section 2.2 is visualized by color coding the base coverage and read alignments according to their mapping class in the data viewers described later. By default, Perfect mappings are displayed in green, Best Match mappings in yellow and Common mappings in red (Fig. 2). This classification coloring is user adjustable.

To facilitate quick exploration of interesting regions, ReadXplorer provides several views on the data. The reference viewer displays all six reading frames, besides the sequence of both strands. The track viewer shows a coverage plot. The double track viewer visualizes the coverage differences between two tracks, and the multiple track viewer combines the coverage of selected tracks in one dataset (Supplementary Fig. S1C). To enhance comparability of tracks, these track viewers are able to normalize the coverage plot separately for each included track. The histogram viewer supplies intuitive exploration of position-specific coverage information. The interactive alignment viewer displays each computed read alignment and colors the mappings according to their mapping quality. Both histogram and alignment viewer simplify the visual identification of SNPs or variation in the data. For paired-end or mate pair data, the read pair viewer shows the pair configuration of all aligned reads. Each pair is color coded in the same manner as the mapping classes: Perfect pairs in green, Distorted pairs in yellow and Single Mappings in red (Supplementary Fig. S2A). The thumbnail viewer is available for direct comparison of the coverage of multiple genomic annotations from multiple datasets at a glance (Supplementary Fig. S2B). The current position is synchronized among all these data viewers for the same reference.

## 3 ANALYSIS FUNCTIONS

In the development of our rich client application, we focused on the implementation of automated analysis functions that enable users to perform laborious tasks for a selected list of tracks in virtually no time. All integrated analysis methods, in particular, rely on the quality classification of the data. All parameters are user adjustable and configured via simple wizards.

Analyses supported by ReadXplorer are SNP and insertion–deletion polymorphism (DIP) detection, a reference feature coverage analysis and RNA secondary structure prediction. Especially for RNA-Seq experiments, we offer differential gene expression analysis, TSS, novel transcript and operon detection as well as RPKM value and read count calculations for each reference feature. The results of all these analyses are displayed to the user in the form of tables that can be sorted by each column, filtered by column values and directly exported into Microsoft Excel files. For an effortless visual assessment, results are additionally highlighted in their corresponding data viewers. Furthermore, the reference position of the currently selected result is centered in each corresponding data viewer.

### 3.1 SNP and DIP detection

SNP and DIP detection is one of the key analyses when sequenced reads are mapped on a closely related reference sequence. Our implemented SNP and DIP detection not only reveals small-scale evolutionary differences but also allows analysis of the resulting functional differences emerging from the polymorphisms already in the results table. The software detects SNPs and DIPs based on a user-definable minimum percentage of variation and a minimum count of mismatching bases in the mappings at the examined position. The results can be examined in detail not only in the alignment or histogram viewer but also in the track viewer (Supplementary Figs S1A and S3). Further, the detected SNPs and DIPs are classified according to their type (substitution, insertion or deletion), their location (intragenic or intergenic) and their effect on coding sequences. Therefore, associated codons and amino acids are shown for intragenic SNPs (Supplementary Fig. S3).

### 3.2 Transcription start site and novel transcript detection

The detection of novel TSSs as well as the verification and correction of already annotated TSS are two of the key analysis features for RNA-Seq experiments. When using a suitable RNA-Seq protocol, such as the selective analysis of primary transcripts (Borries *et al.*, 2012) or a 5′ RNA adapter ligation (Pfeifer-Sancar *et al.*, 2013), the mapped reads can be analyzed for TSSs.

We have developed and implemented a novel method that detects TSSs for each neighboring genomic position pair. It mainly relies on two parameters balancing each other. A TSS is only detected if enough reads start at the second position of the pair and the increase of coverage in percentage from the first to the second position of the pair is high enough (Supplementary Fig. S5A).

Both parameters are user adjustable but can also be automatically estimated for each track. In addition, a specialized treatment for low-coverage regions has been implemented. The automated parameter estimation only emits statistically significant positions as TSS, which satisfies both of the following two empirically chosen criteria:

1. The absolute number of read starts is in the upper 0.0025 quantile of all other read start counts for all positions in the track.
2. The percentage of coverage increase is in the upper 0.0025 quantile of all other coverage increase percentages in the track.

By introducing the second parameter, we reject positions in areas of already high coverage, where the total number of read starts would lead to a detected TSS. These positions are now rejected if the increase only accounts for a low increase in percentage.

Our parameter choice is also suitable for 5′-enriched RNA-Seq datasets because the underlying distribution takes into account each n − 1 pairs of neighboring positions in a genome of size n.

Prokaryotes are known to exhibit approximately one gene per kb of genome size (Rogozin *et al.*, 2002). With our parameter choice, we allow for 2.5 genes per kb of genome size for each of the two parameters. Thus, there is enough room for both parameters to balance each other, and in practice, they have shown to be stringent (see Chapter 4).

In some cases, the coverage increases in steps at a TSS. To consider this case, we only report the best scoring position within each 3 bp window as TSS.

Our TSS analysis also supports the detection of novel transcripts. Therefore, we list the nearest annotation starting in the right direction in a 1000 bp window around a TSS. In case a TSS is detected without a neighboring annotation, it is marked and a novel transcript is suggested, starting at the TSS and ranging up to the position at which the coverage drops below a user-defined threshold (Supplementary Fig. S5A).

Furthermore, we can detect *trans*-encoded as well as *cis*-antisense transcripts and microRNAs (miRNAs). Additionally, highly conserved miRNA target sites can be detected by exploiting the fact that reads match to their origin as well as to their target site.

Because the detection algorithm does not need any annotations, our method is well suited as a starting point to identify novel transcripts in references without any genomic annotations. Afterward, they can be filtered manually and verified in the laboratory.

Additionally, we have implemented highlighting of start and stop codons and their open reading frames in the reference. This facilitates instant comparison of RNA-Seq coverage with potential transcripts.

As validation, we have compared a set of 223 known TSS from *Corynebacterium glutamicum* in an RNA-Seq dataset using the automatic parameter estimation (see Supplementary Table S1). Our method was able to detect 78.87% of the TSS, which show at least the minimal required coverage increase. The remaining 21.13% do not satisfy the stringent automatic analysis parameters, either because of a low number of read starts or a stepwise coverage increase over multiple neighboring genome positions in this dataset. By relaxing the parameters manually, these TSS could also be identified.

### 3.3 Differential gene expression analysis

Transcript quantification by RNA-Seq offers a high-resolution insight into expression levels. In comparison with other methods, like microarrays, it has been shown that RNA-Seq delivers far more accurate measurements (Wang *et al.*, 2009) for differential gene expression analysis. In ReadXplorer, we have integrated two widely used publicly available tools for differential gene expression analysis: DESeq (Anders and Huber, 2010) and baySeq (Hardcastle and Kelly, 2010). Both are written in R and available as R packages via Bioconductor (http://www.bioconductor.org). Both tools assume a negative binomial distribution to deduce statistically significant differences in read count for the same feature under different environmental conditions. DESeq uses variance and mean calculations linked by local regression, whereas baySeq is based on the complex mathematical model of an empirical Bayes approach. Additionally, we have implemented a novel differential gene expression approach, the so-called *Express test*. Our goal was to implement an ultra-fast method without extensive statistical tests to complement the other two tools. This, of course, might come at the expense of accuracy, but it allows rapid insight into the data (see Supplementary Fig. S11). The Express test is limited to two conditions and calculates normalized gene expression values and a confidence value but does not perform a statistical test for differential expression. If we have two conditions *A* and *B* with *n* and *m* replicates, we have the samples $\left\{a_{1},a_{2},...,a_{n}\right\}$ and $\left\{b_{1},b_{2},...,b_{m}\right\}$. wϵ{n,m} denotes the number of samples of one of the two conditions. The read count of the same *k* regions $R_{i}$ with iϵ{1, 2, ..., k} is analyzed in each of these samples. $R_{i,x}$ denotes all samples of one region belonging to a condition *X*, where Xϵ{A,B}. Mean and variance are then defined as

$$mean\;{\text{ (R}}_{i,x})\text{ = }\bar{R_{l,x}}=\frac{\sum_{v=1}^{w}R_{v,i,x}}{w}$$

$$variance(R_{i,x})=\frac{\sum_{v=1}^{w}{\left(R_{v,i,x}-\bar{R_{l,x}}\right)}^{2}}{w-1}$$

Additionally to mean and variance, the ratios

$$\frac{{mean\text{(R}}_{i,A}\text{) }}{{mean\text{(R}}_{i,B}\text{) }}\;\text{and }\;\frac{{mean\text{(R}}_{i,B}\text{) }}{{mean\text{(R}}_{i,A}\text{) }}$$

are computed. If a mean value in the denominator is zero, it is replaced by one. In the last step, a confidence value $C_{i}$ is computed for each $R_{i}$ with $mean{\text{ (R}}_{i,x}\text{) > 0}$ by the following equation:

$$C_{i}=-\log_{10}\left[\frac{1}{2}\left(\frac{{variance\text{(R}}_{i,A}\text{) }}{{mean\text{(R}}_{i,A}\text{) }}\text{ + }\frac{{variance\text{(R}}_{i,B}\text{) }}{{mean\text{(R}}_{i,B}\text{) }}\right)\right]$$

For mean values of zero, $C_{i}$ is set to −1. A high $C_{i}$ value can only be obtained by $R_{i}$ with a small variance. Hence, high ratio and confidence values indicate differential gene expression of the associated genomic region $R_{i}$. To account for inhomogeneous sequencing depth between the samples, the Express test offers a normalization of the results. The normalization ratios can be computed based on all regions or based on a list of regions (e.g. housekeeping genes) adjustable by the user. The advantage of the Express test is that it is completely implemented in Java. Therefore, it also works if R is not available on the current machine. All three tools can be configured within ReadXplorer by a simple wizard. The count data to be analyzed are then prepared by ReadXplorer, and for DESeq and baySeq, transferred to R by using rJava (http://www.rforge.net/rJava/). After execution, the differential gene expression result tables are automatically reimported into ReadXplorer (Supplementary Fig. S6) and interactively presented to the user. Based on the results, typical plots like MA plots (log2 fold change (M) plotted against normalized mean expression (A)) can be generated. These plots are interactive; thus, a selected point in a plot links back to the annotation it represents in the viewers (Supplementary Fig. S7). This feature allows effortless visual assessment of the original data on which the differential gene expression results are based.

### 3.4 Genomic feature coverage and general coverage analysis

Our two coverage analyses empower an experimenter to detect all reference features or reference intervals that show exceptional characteristics in terms of their coverage. They allow either listing of all features or intervals that are covered to a high extent by mapped reads or, on the contrary, that are not covered by a satisfactory amount of reads.

These analyses are useful for studies of both RNA-Seq and resequencing datasets. For RNA-Seq experiments, they facilitate the identification of genes or intervals that exhibit a certain minimum coverage or that are not expressed or covered at all. For resequencing experiments, the feature coverage analysis enables exploration of the set of common or divergent features between the reference and its tracks and among the tracks (Supplementary Fig. S9). The general coverage analysis allows identification of regions that could not be sequenced (Supplementary Fig. S10) or show a significantly higher than the average coverage.

### 3.5 RPKM and read count calculation

This analysis offers filtering of reference features in two ways: either by minimum and maximum RPKM value (total exon reads per million mapped reads per kilobase of exon model) (Mortazavi *et al.*, 2008) or simply by minimum and maximum raw read count (Supplementary Fig. S8). The RPKM option is well suited for RNA-Seq data to identify genes with a certain expression level, whereas the read count option is applicable for both RNA-Seq and resequencing data to explore the read counts of reference features.

### 3.6 Operon detection

In prokaryotes, the annotation of operons (co-transcribed sets of genes resulting in a single polycistronic messenger RNA) is of high importance (Westover *et al.*, 2005). Operons can be identified in RNA-Seq datasets by closely analyzing the coverage. In terms of coverage, the most significant evidence in a track for two genes belonging to an operon is observed if these two neighboring genes are connected by a reasonable amount of unique single reads overlapping both genes. If the distance between both genes is too large to be spanned by single reads, the minimal coverage in the interval between both genes can be taken into consideration.

In ReadXplorer, two neighboring genes (protein-coding sequences or RNAs) are assigned to an operon if the number of observed reads spanning the two genes is higher than a user-defined threshold. This threshold varies from track to track among others depending on the background noise (Supplementary Fig. S4).

### 3.7 RNA folding

The folding of RNA sequences, for example in untranslated regions, can reveal many structural and functional properties. Thus, we have included the ability to either fold a read alignment or a sequence of interest chosen from the reference sequence by querying the online service of RNAfold (Hofacker, 2003; Hofacker *et al.*, 1994) coupled with the graphical output of RNA Movies (Evers and Giegerich, 1999; Supplementary Fig. S1D).

## 4 RESULTS

ReadXplorer is already widely in use at several sites. Some of the conducted experiments and their main results generated by ReadXplorer are explicated in the following.

During a pan-genome analysis of 20 *Pseudomonas aeruginosa* strains (Hilker *et al.*, Unpublished data), ReadXplorer was successfully used to analyze the SNPs and DIPs of each strain compared with the reference strain PAO1 (Stover *et al.*, 2000). Our read classification has already proven useful here. Many possible SNPs would have been missed (e.g. 1300 possible SNPs, of which >900 are intragenic, for the most divergent strain) if only uniquely mapped reads had been considered. Instead, we relied on all Perfect and Best Match mappings of the reads but excluded all Common Match reads. Even when all 20 datasets are opened simultaneously, the performance and responsiveness of ReadXplorer is convincing. For example, it only used 740 MB of RAM when displaying the coverage of a 76 kb interval containing ∼200 000 reads per strain for each of the 20 genomes and—in parallel—calculating SNP detection results for these strains.

Further, the capabilities of the feature coverage analysis have been demonstrated during investigation of known regions of genomic plasticity (RGP). By using this analysis, it was possible to exactly determine the appearance of each RGP among all strains in percent and in base pairs (Hilker *et al.*, Unpublished data).

The effective handling of medium sized eukaryotes has been demonstrated during an in-house collaboration for the SNP and DIP analysis of three closely related *Chlamydomonas reinhardtii* (Merchant *et al.*, 2007) strains in a resequencing experiment (data not shown).

The TSS and operon detection, as well as the differential gene expression analysis tools have effectively been applied to experiments on different *C.glutamicum* strains (e.g. Mentz *et al.*, 2013; Pfeifer-Sancar *et al.*, 2013). The integrated differential gene expression toolkits delivered similar results as corresponding microarray experiments (data not shown). The differences in both assays might be induced by the higher resolution and flexibility of RNA-Seq in comparison with microarrays.

Additionally, we have performed an assessment of the performance of all available analysis functions in ReadXplorer. The results of this benchmark are shown in Supplementary Table S2. A comparison of the software features implemented in ReadXplorer to the five other tools mentioned in Section 1 is also given in the Supplementary Material (Supplementary Table S3).

## 5 CONCLUSION

ReadXplorer is an interactive visualization and analysis software designed to simplify and accelerate NGS data processing. It introduces a novel classification approach for sequencing reads, which has already successfully aided several analyses, and provides corresponding color-coded visualizations that allow comprehensive exploration and evaluation of this kind of data. One visualization, the so-called double track viewer, features superimposition of coverage and, thereby highlights all differences between two different tracks. Combined data analysis is simplified by a multiple track viewer by adding up coverage from different tracks and using ReadXplorer's analysis functions on such a combined track. Highlighting of start and stop codons and their coding sequences facilitates instant comparison of RNA-Seq coverage with potential transcripts. ReadXplorer already integrates several automated RNA-Seq analysis functions including differential gene expression analysis, detection of TSS, novel transcripts, operons as well as calculating RPKM and feature read count values. Further, it allows SNP and DIP detection, RNA secondary structure prediction and analyzing the coverage of reference features and the whole reference sequence.

By providing all these different analysis functions from one source, there is no need to install and handle several different tools. Thus, the learning curve for users is shortened, and tedious conversion of different output from different tools is no longer required. Furthermore, the modular software composition and plug-in framework enable simple integration of additional highly specialized modules by other developers. Therefore, selected ReadXplorer features have already been implemented by students within the framework of a 2 week course without any prior knowledge of ReadXplorer.

## Supplementary Material

Supplementary Data
