# Supplementary material for: ReadXplorer—visualization and analysis of mapped sequences
Source: Bioinformatics. 2014 Apr 30;30(16):2247–54. doi: 10.1093/bioinformatics/btu205 (PMC4217279; doi:10.1093/bioinformatics/btu205)
Supplement: Supplementary Data [file supp_30_16_2247__index.html]

ReadXplorer—visualization and analysis of mapped sequences — Supplementary Data 

# ReadXplorer—visualization and analysis of mapped sequences

## Supplementary Data

file

**Files in this Data Supplement:**

- Supplementary Data - zip file
