# Supplementary material for: ReadXplorer—visualization and analysis of mapped sequences
Source: Bioinformatics. 2014 Apr 30;30(16):2247–54. doi: 10.1093/bioinformatics/btu205 (PMC4217279; doi:10.1093/bioinformatics/btu205)
Supplement: Supplementary Data [file supp_btu205_2014-03-27-Supplement.zip › 2014-03-27-Supplement.docx]

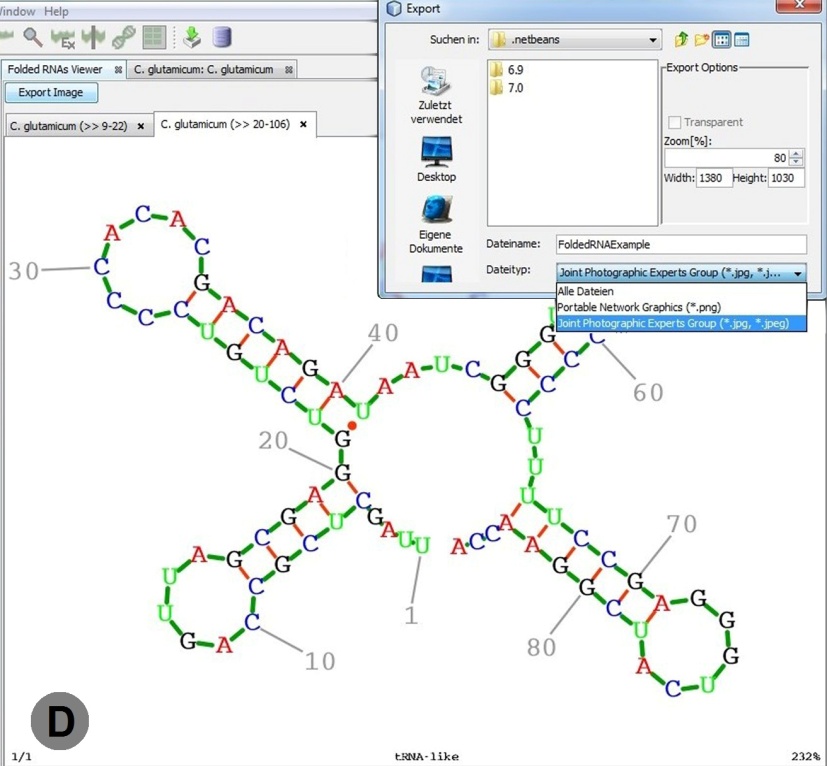

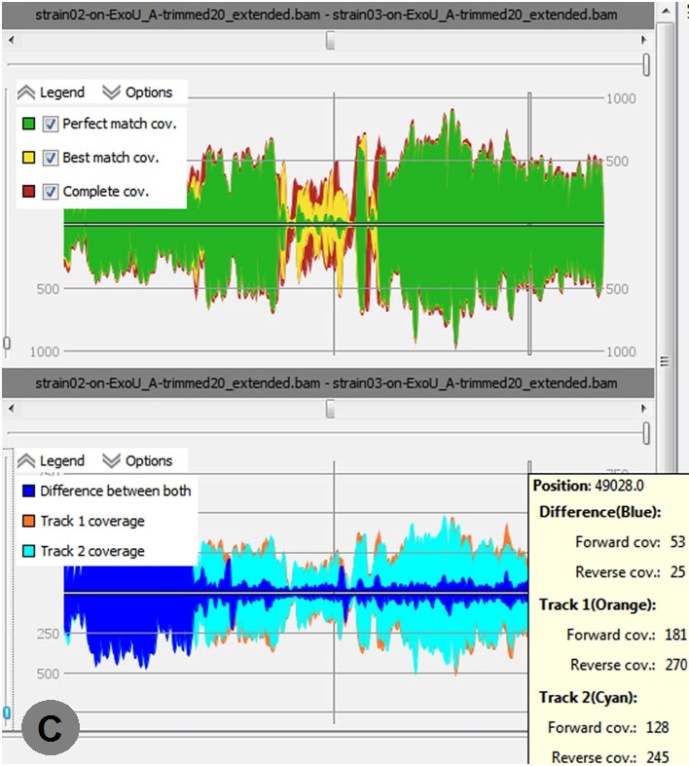

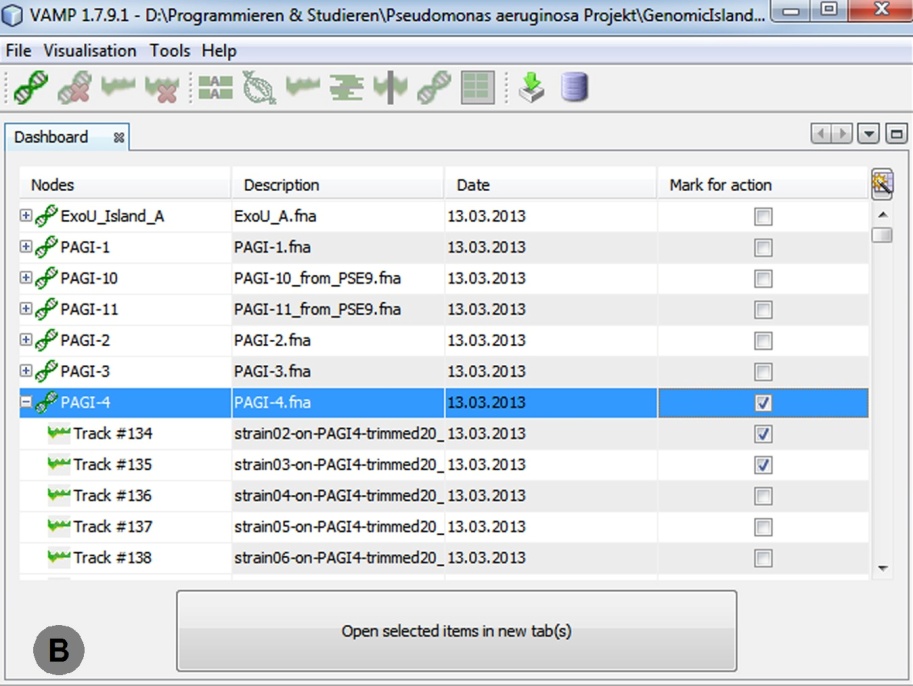

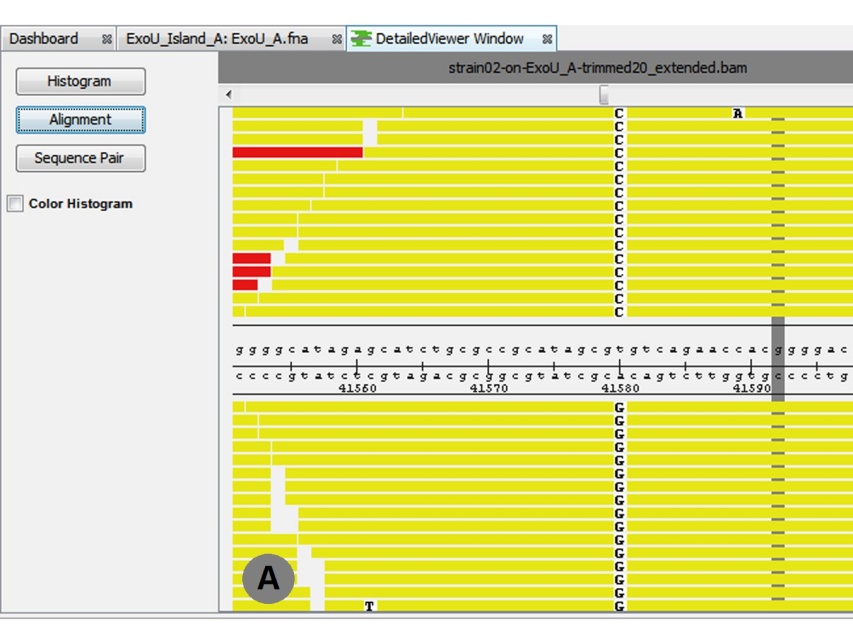


Figure S1:

A) Alignment Viewer: The alignment viewer displays each computed read alignment and colors the mappings according to their quality classification in green (Perfect Match), yellow (Best Match) or red (Common Match). Details about each mapping are accessible in a tooltip, which appears when hovering a mapping. The Alignment Viewer is well suited to visually identify potential SNPs and DIPs like at the centered position.

B) Dashboard: The dashboard visually organizes the data of a project and allows to quickly select and view data sets.

C) Multiple and Double Track Viewers: The Double Track Viewer (bottom) visualizes the coverage differences between two tracks and the Multiple Track Viewer (top) combines the coverage of selected tracks in one data set. To ease comparability of tracks, these track viewers are able to normalize the coverage plot separately for each included track. In the example the same two *P. aeruginosa* RGP data sets (see Chapter 4) are overlayed in the Double Track Viewer and combined in the Multiple Track Viewer. In the double track viewer in the left part track one has no coverage, thus the large blue area, depicting mapping differences, can be observed. Therefore either this area is not present in the track one strain, or it could not be sequenced correctly, while it is present in the track two strain. To the right of that area both tracks show similar coverage values and the coverage of track one is generally slightly higher (orange).

D) RNA secondary structure prediction: The cloverleaf structure is predicted for a selected putative tRNA sequence of *C. glutamicum*. The figure also shows the save dialog for the image.


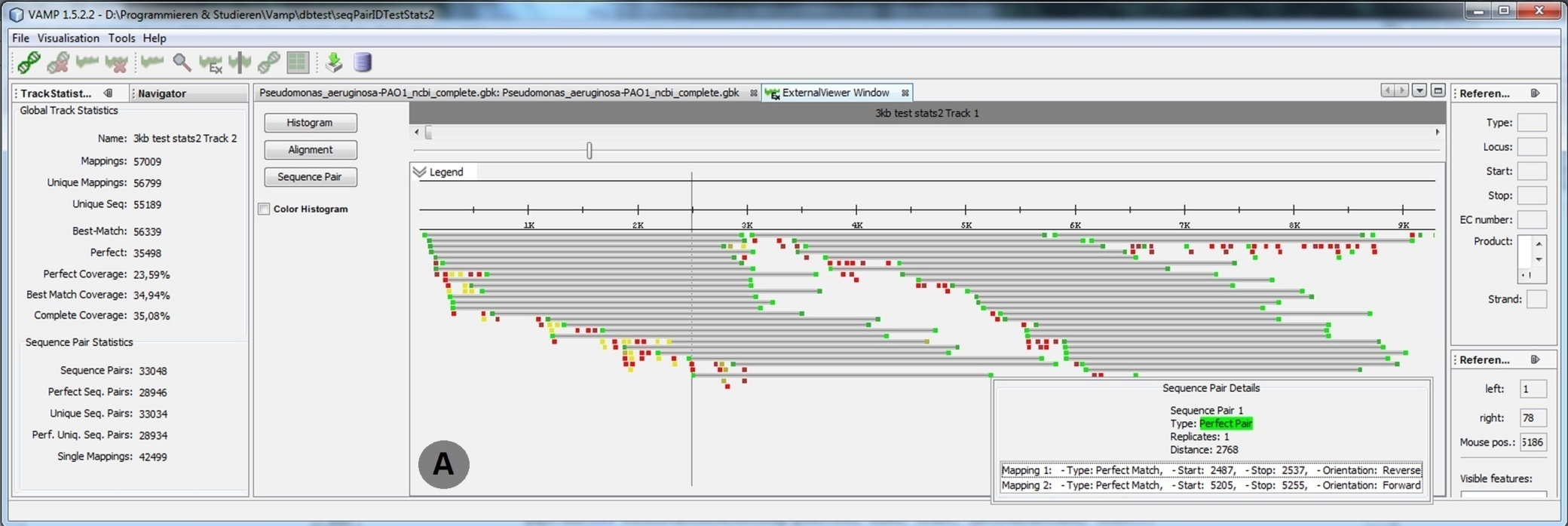


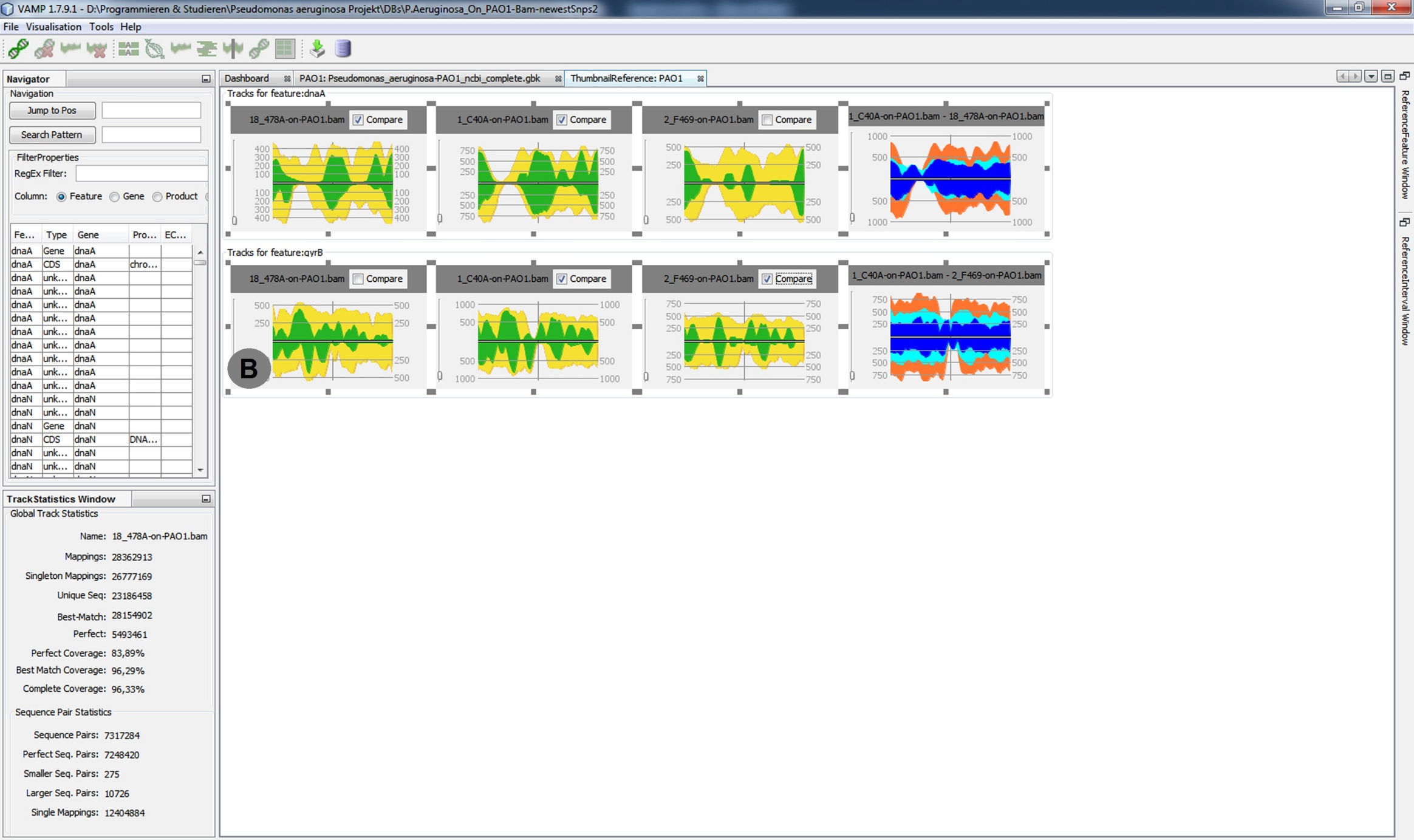


Figure S2:

A) Read Pair Viewer: The Read Pair Viewer shows the pair configuration of all aligned reads for paired end or mate pair data. Perfect pairs are displayed in green, Distored pairs in yellow and Single reads in red. This color coding is user adjustable. By clicking on a mapping, additional information is displayed about the pair and repetitive regions can easily be scanned by selecting, and thus jumping to, other mappings of the pair from the list of mappings in the popup.

B) Thumbnail Viewer: The Thumbnail Viewer is available for direct comparison of the coverage of multiple genomic features from multiple data sets at a glance. In this example the coverage of two features is compared among three P. aeruginosa tracks. The overall coverage pictures are generally similar, but in the two double track viewers on the right side it can immediately be observed that in both comparisons the coverage of track one (orange) is much higher than the coverage of track two.


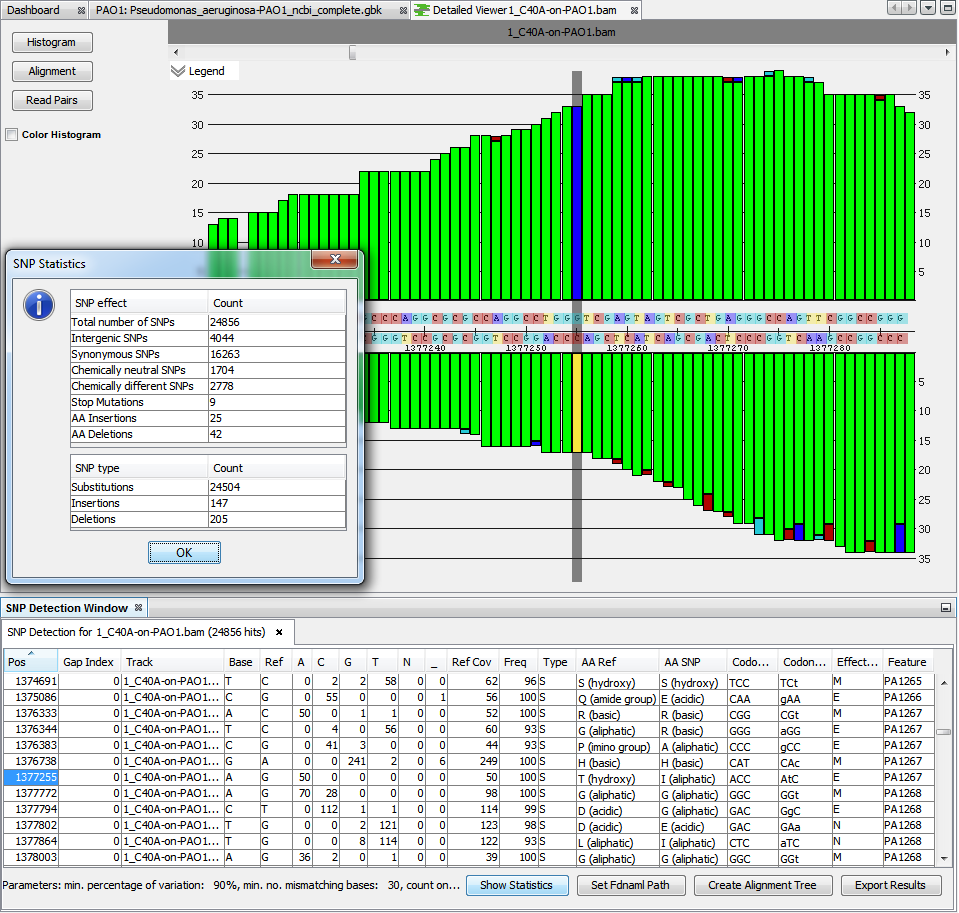


Figure S3: SNP and DIP Detection: A SNP and DIP detection result for a *P. aeruginosa* strain with a minimum percentage of variation of 90 percent and at least 30 mappings with the same mismatching base at the examined position. The selected position in the result table is a substitution, which is easily identified as SNP in the Histogram Viewer. The result table shows that it is located within the gene "PA1267" and leads to a chemically different amino acid within that gene's coding sequence ("E" in the "Effect on AA" column means "chemically different"). The SNP statistics window in the middle displays the overall statistics of the SNP and DIP detection for this strain.


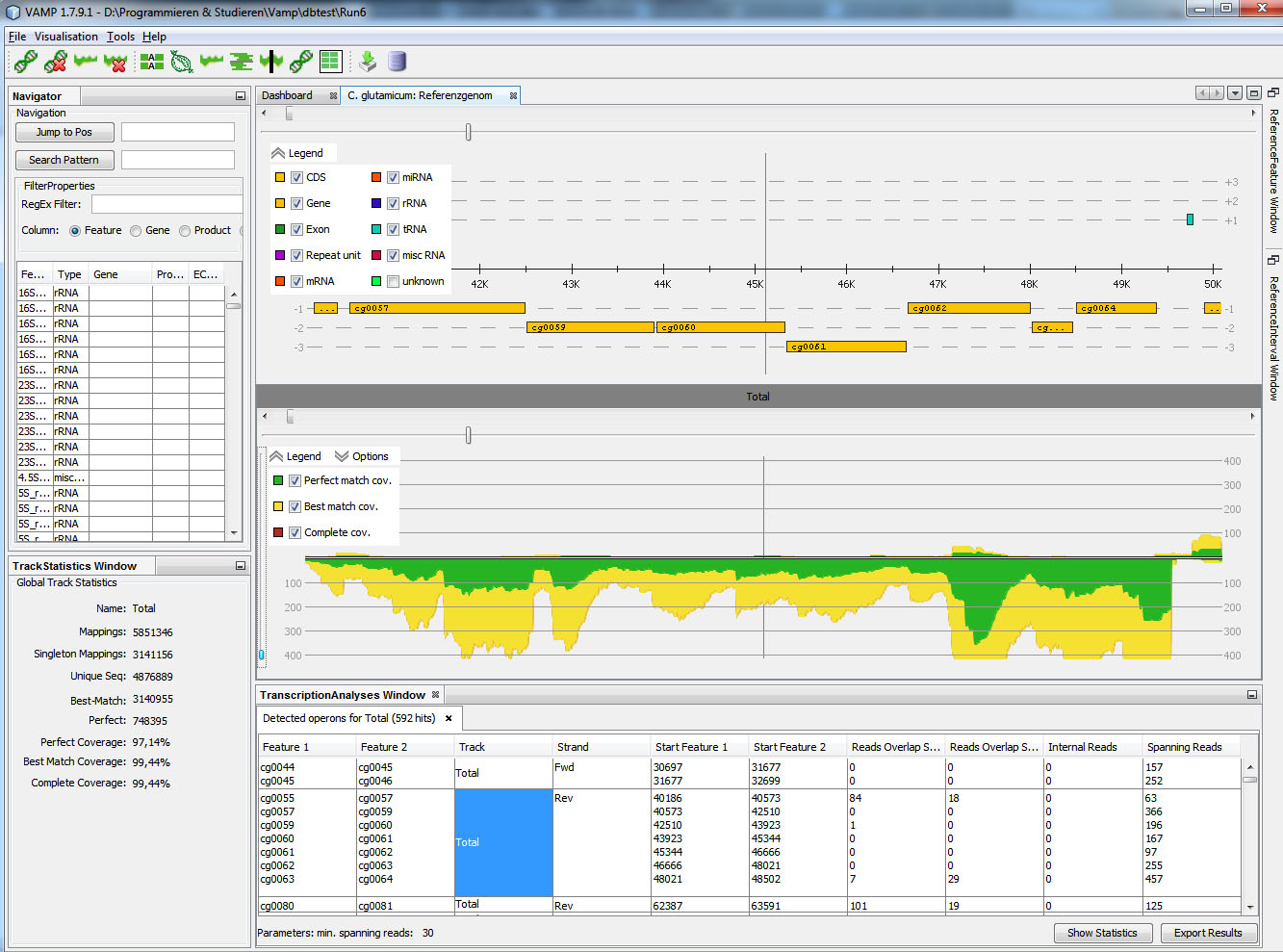


Figure S4: Operon Detection: The operon detection has detected 592 operons with a minimum number of 30 spanning reads in the shown *C. glutamicum* RNA-Seq track. The selected putative operon interval is displayed in the reference and track viewers and the coverage can be closely analyzed. These results can then aid further analysis of interesting predicted operons in the laboratory.


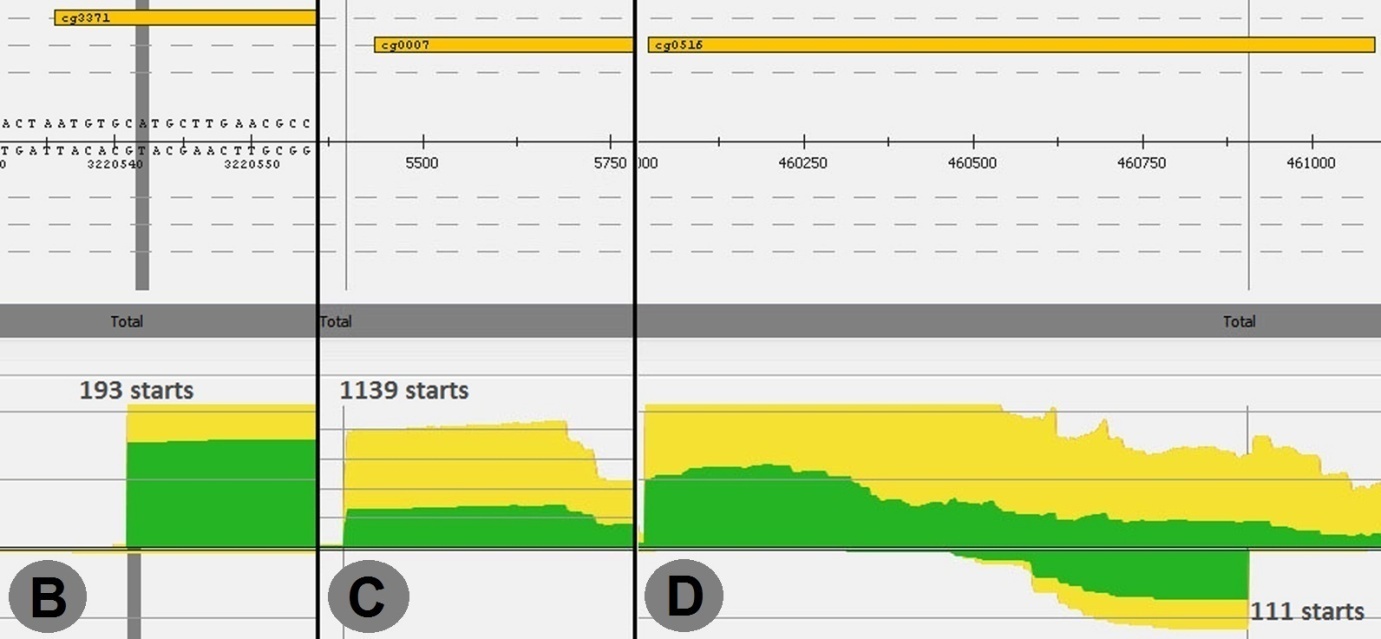

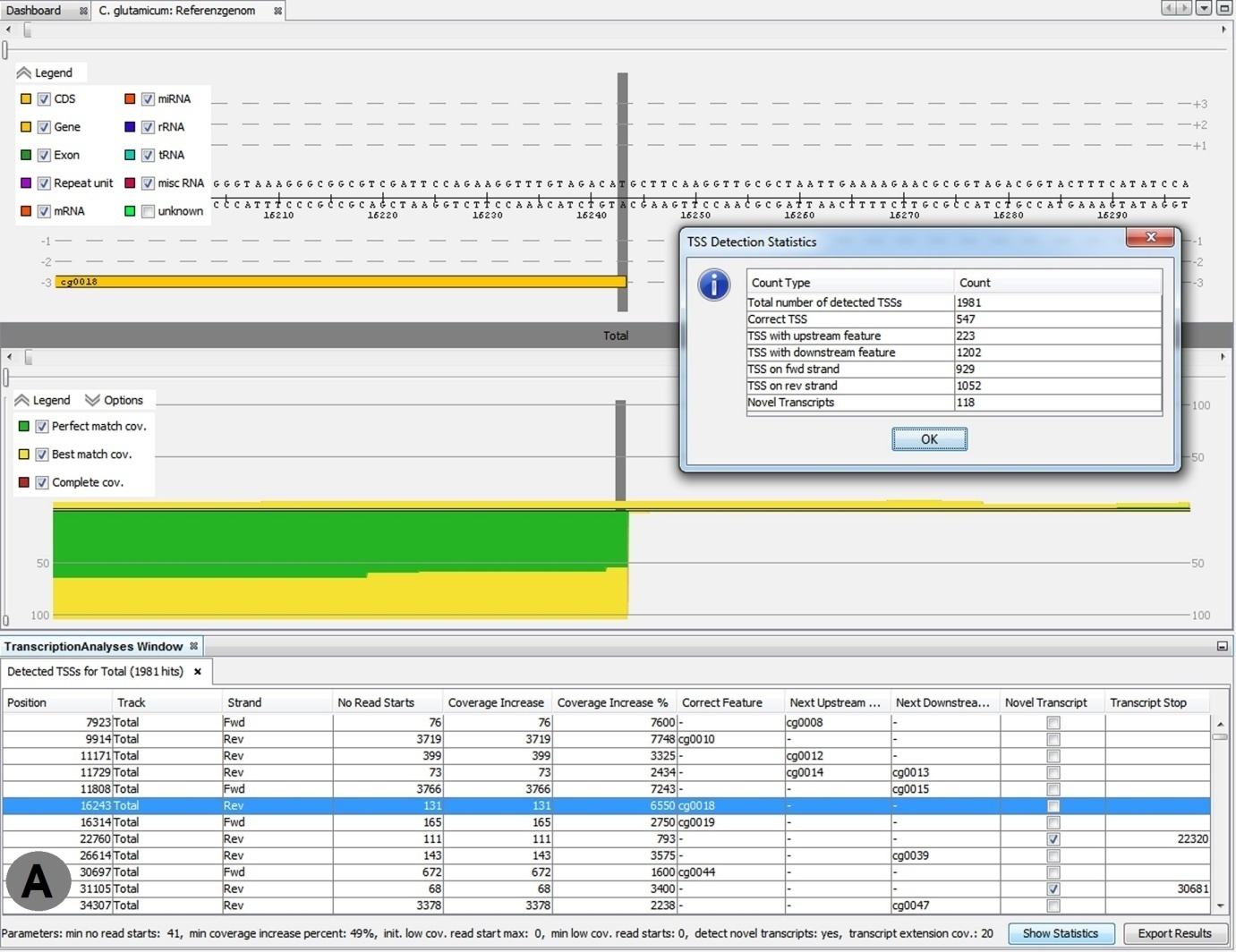


Figure S5:

A) TSS Detection: In this example 1901 TSS for a *C. glutamicum* RNA-seq track were predicted for the parameters listed below the result table. Also the novel transcript detection was enabled. Thus, all novel transcripts can be identified by the check in the second last column of the table. The selected TSS position, which is centered in the Reference and Track Viewer is located directly at the start of a gene annotation. In this case a leaderless gene has been found, whose annotation is supported by the TSS detection.

B) Wrongly annotated start: The selected TSS position is located downstream of the annotated transcript start. In this case the annotation could be refined, since the result is a very sharp ascent of 193 reads starting at the TSS.

C) Unannotated 5'UTR: The TSS of the shown gene is located upstream of the annotation. Thus, this gene has a 5'UTR, which we have detected in this case with 1139 reads starting at the TSS.

D) Novel antisense transcript: Using the novel transcript detection, we have found a novel antisense transcript to the annotated and expressed gene on the forward strand. It starts with 111 reads at the TSS.


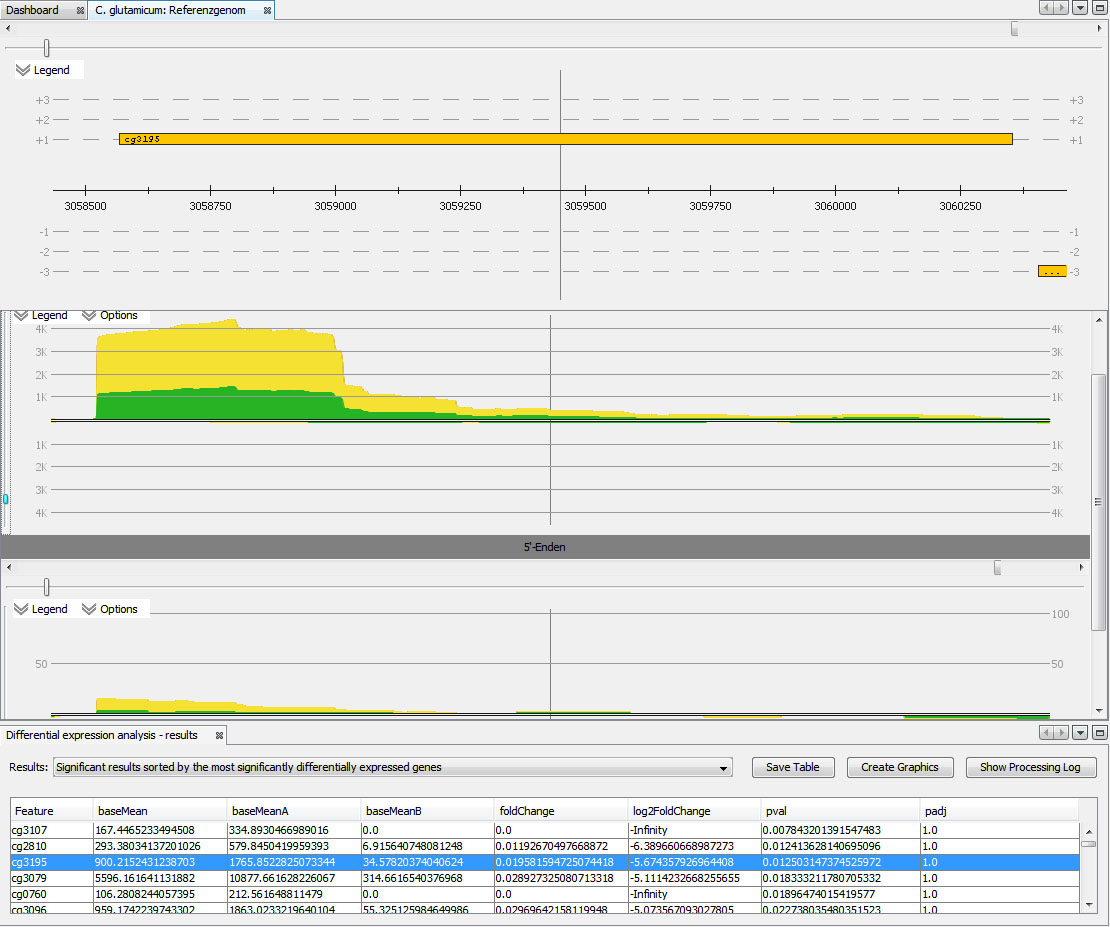


Figure S6: Differential Gene Expression Analysis: In this example we ran DESeq from within ReadXplorer on a *C. glutamicum* RNA-Seq experiment. The selected and displayed gene is detected as one of the most differentially expressed genes. It clearly has a much higher expression rate in track one (a coverage at the start around 4000 mappings) than in track two (a coverage at the start around 20 mappings).


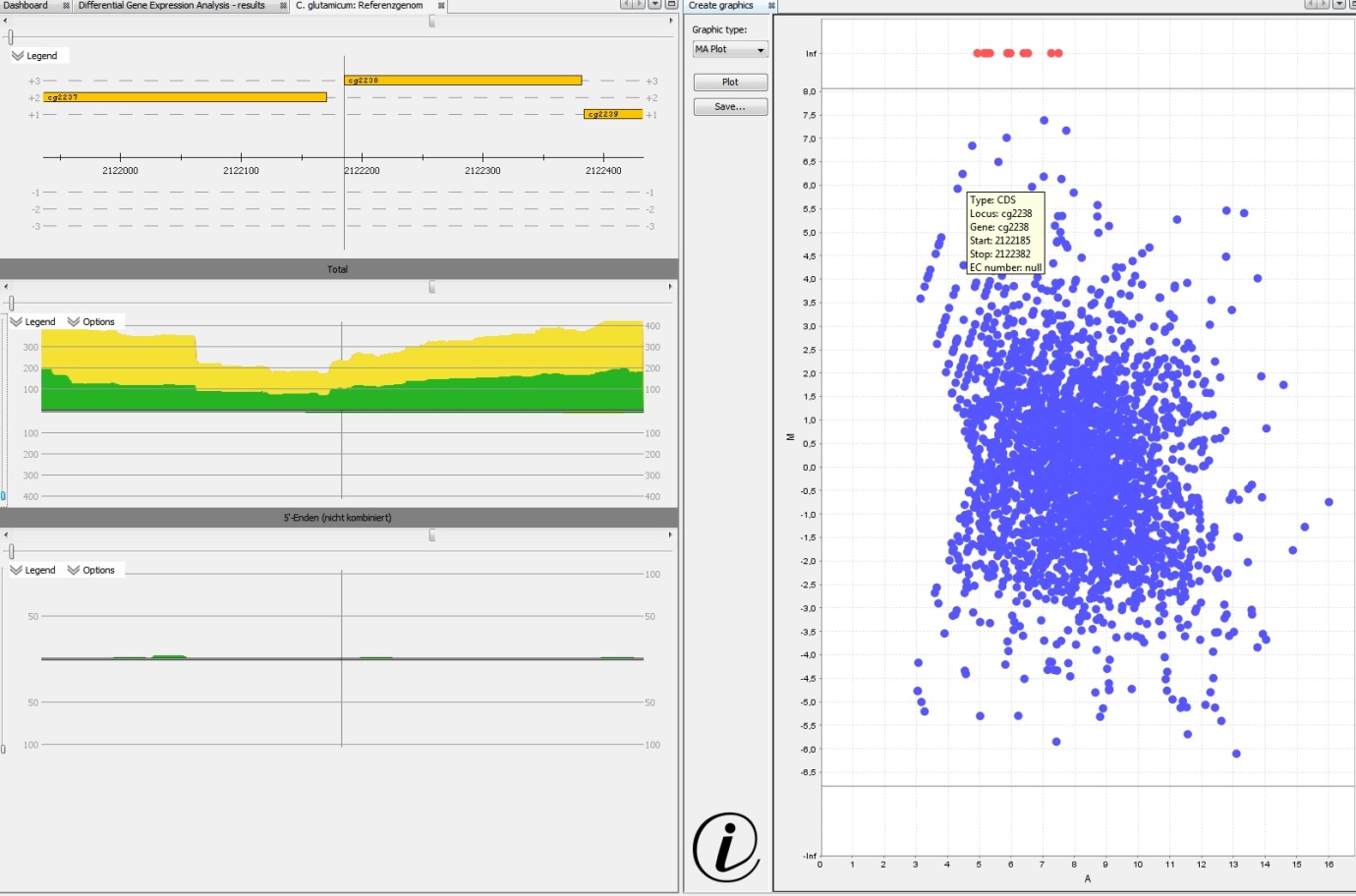


Figure S7: Differential Gene Expression Analysis Plots: The interactive MA plot allows to easily access the underlying experimental data for each gene. Each dot represents a gene and the farther away a gene is located from zero on the Y-axis, the more differentially expressed the gene is. When clicking on a dot, the corresponding gene is centered in all synchronized data viewers. In the example, we can directly compare the actual coverage of gene cg2238 for both conditions.

**
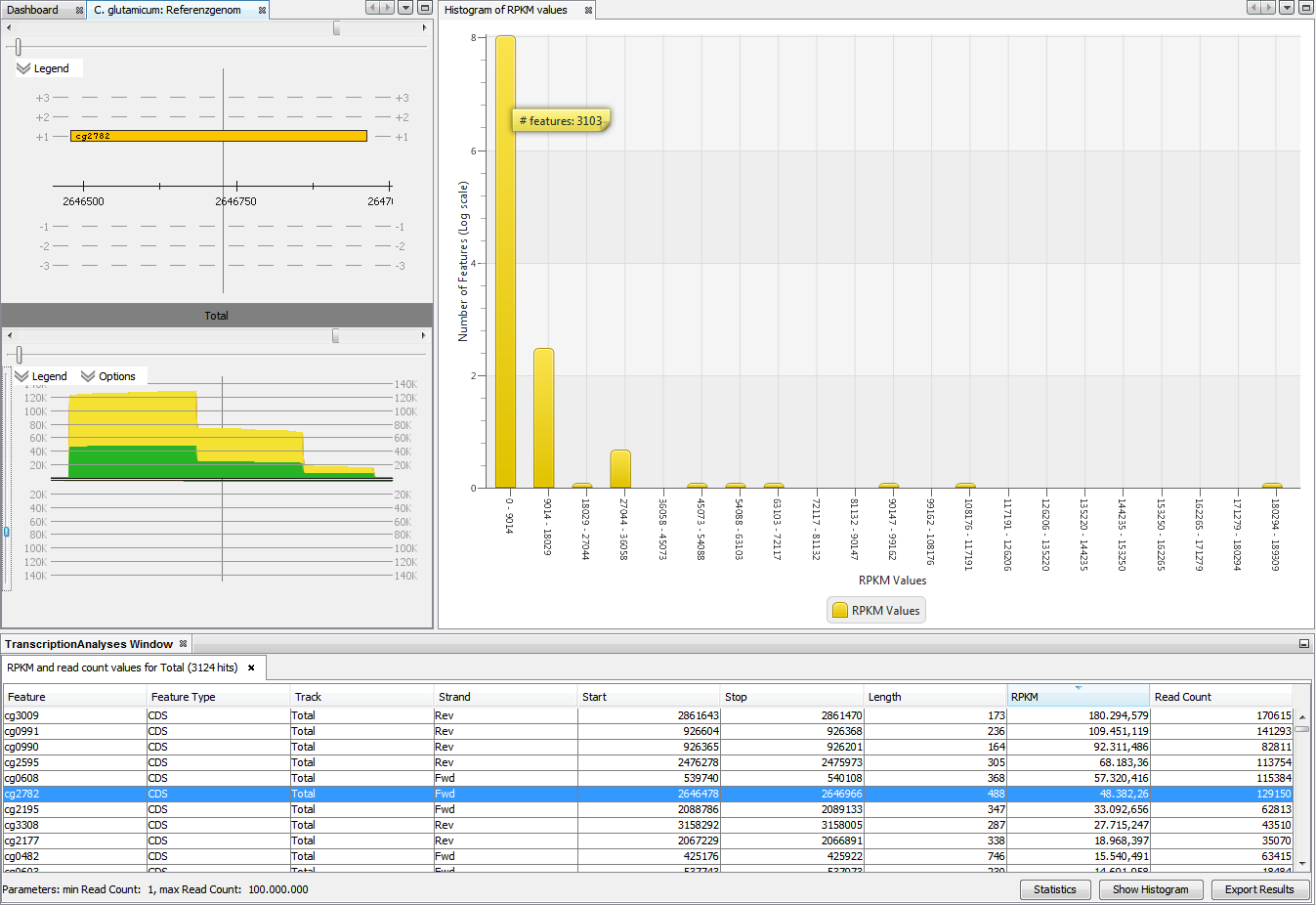
**

Figure S8: RPKM and Read Count Analysis: This analysis calculates the RPKM and raw read count values for all genomic features and omitted all results, which had a raw read count value lower than 1 and higher than 100.000.000. The histogram shows the distribution of RPKM values among all reference features on log scale. The actual amount of features belonging to a bar in the histogram is shown in the tooltip. The tooltip appears, when the mouse hovers above a histogram bar. By left-clicking on the "cg2782" entry in the result table, the synchronized viewers immediately focus on the selected genomic feature, and we can directly investigate the coverage.


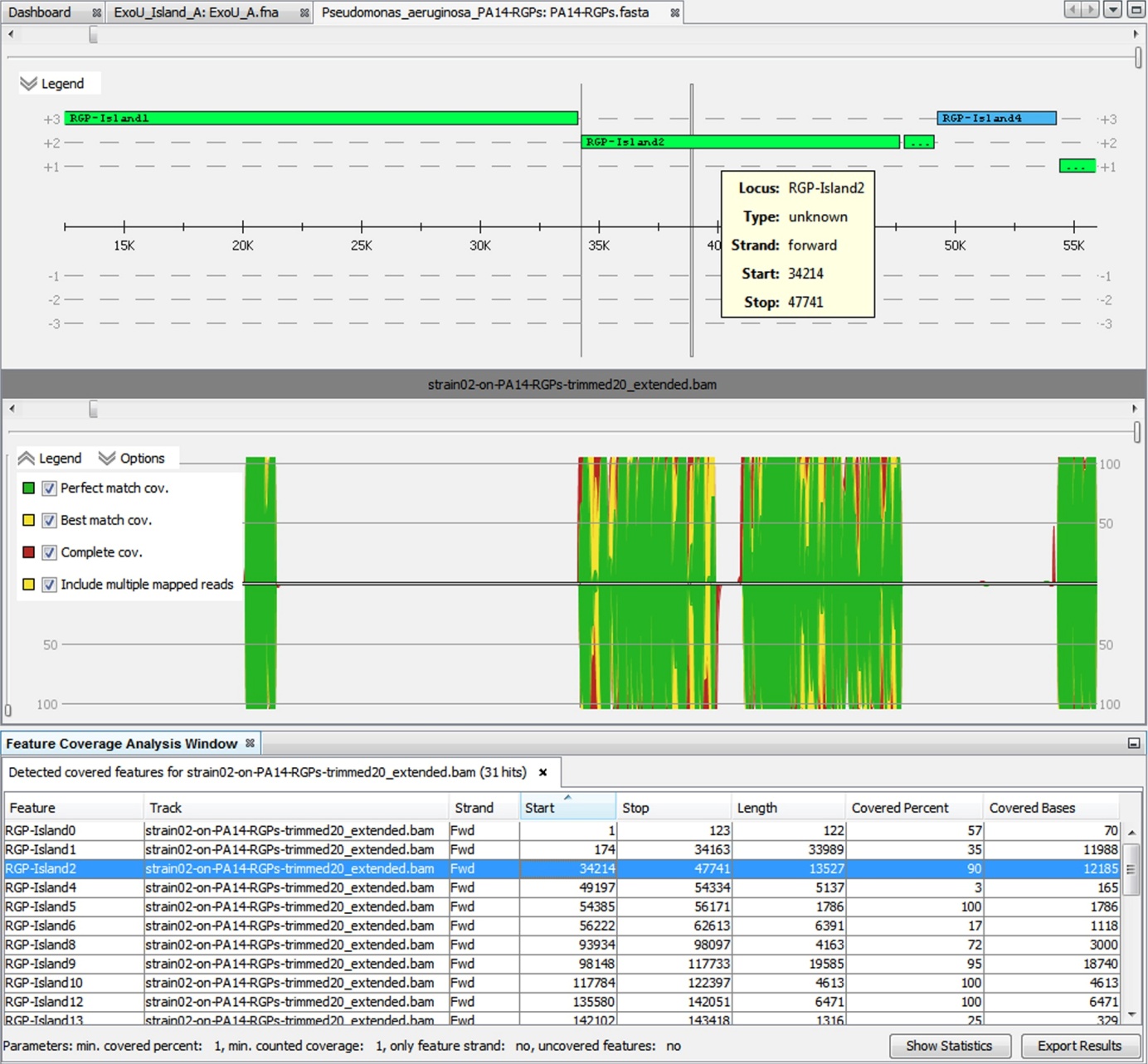
Figure S9: Feature Coverage Analysis: This analysis outputs all genomic features of interest, whose coverage is larger or equal to the minimum covered percent threshold. Only positions with a coverage larger or equal to the minimum counted coverage parameter are counted as covered. The analysis can treat each strand separately or combine the coverage of both strands. Just like the General Coverage Analysis, this analysis offers a second mode, which can detect all features with a coverage not satisfying the given parameters. In the example, the coverage of several regions of genomic plasticity (RGP) from *P. aeruginosa* PA14 was analyzed. This analysis allowed an effortless determination of the appearance of each RGP among all investigated strains.


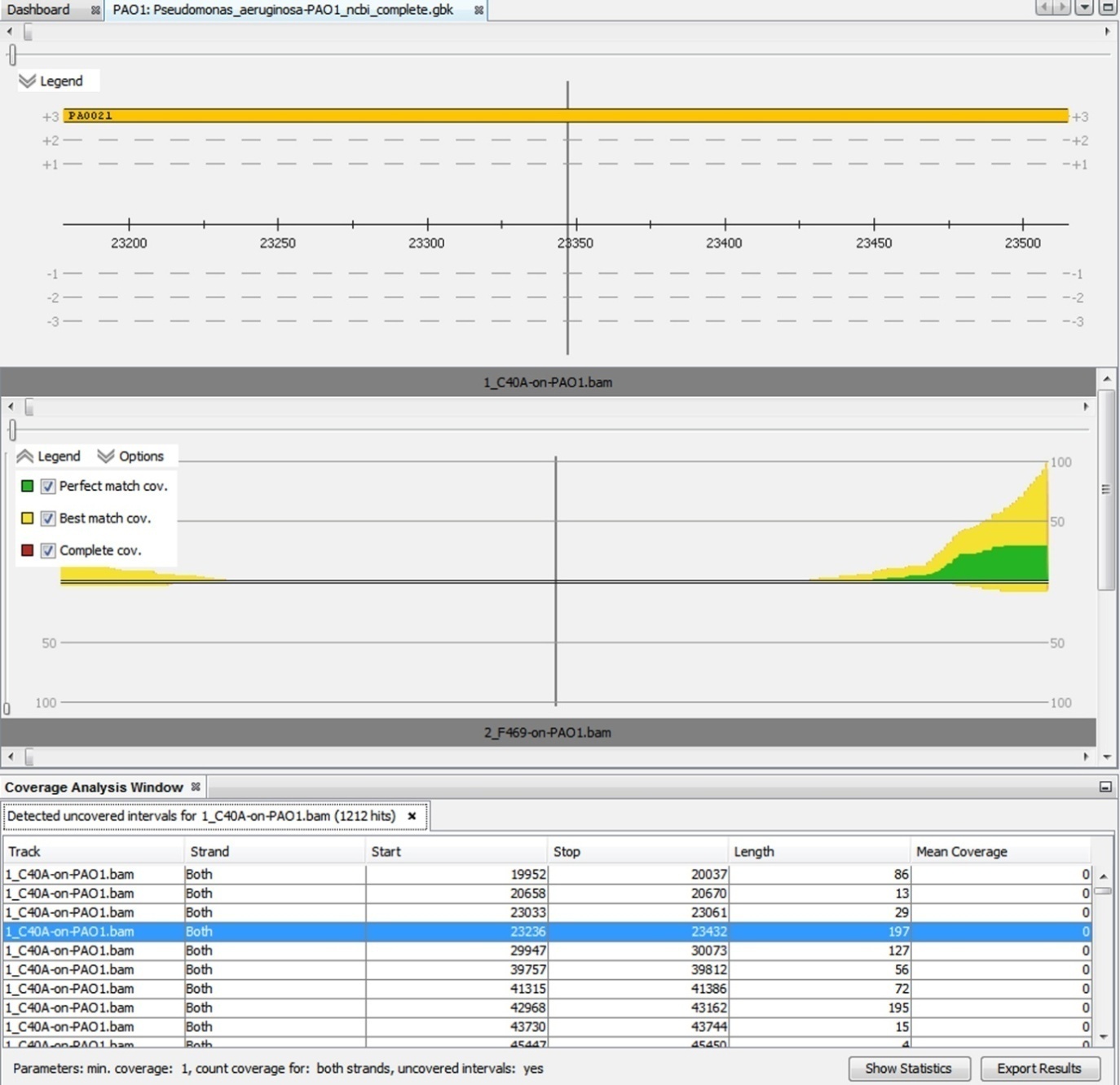


Figure S10: General Coverage Analysis: This analysis was performed to identify all regions (intervals) within the *P. aeruginosa* strain C40A, which do not have any coverage in this genome re-sequencing experiment. Thus, the "uncovered intervals"-mode was chosen and the coverage of both strands was combined. The minimum coverage parameter was set to 1, in order to identify all regions with coverage less than 1. All identified regions either could not be sequenced, or are missing in C40A. In the opposite "covered intervals"-mode, all regions can be detected, which have a coverage larger or equal to the given minimum coverage threshold.

Table S1: Analysis of 223 known *C. glutamicum* TSS. The table lists the detailed result of a comparison of 223 known TSS with an RNA-Seq data set obtained from *C. glutamicum.* 194 of the 223 were manually identified in the analyzed data set. 29 TSS could not be observed, as there is no increase in coverage or read starts in the neighborhood of the expected genome position. 78,87% of the remaining 194 TSS were identified by our novel TSS detection using the stringent automatic parameter estimation. For 13 TSS, an alternative start position has been detected in the analyzed data set in a distance between 13 and 45 bases up- or downstream of the expected TSS position. The 13 expected positions did not show any signs of a TSS in this data set. The lower part of the table apportions the undetected TSS according to their properties. Internal means, that the start is observed within an expressed operon. Stepwise means, that there is an observable amount of read starts/coverage increase around the analyzed position, but it is divided into multiple positions and thus, cannot be detected by our method. The undetected TSS can also be identified by manually choosing and relaxing the analysis parameters, depending on the user needs.

| **# TSS** | **Percentage** | **Description** |
| --- | --- | --- |
| 223 | 100.00% | All TSS |
| 29 | 13.00% | Absent TSS in analyzed data set |
|  | | |
| **Distribution of detectable TSS** | | |
| 194 | 100.00% | Detectable TSS in analyzed data set |
| 140 | 72.16% | Detected TSS |
| 13 | 6.70% | Alternative TSS in 13-45 bp distance |
| 153 | 78.87% | Total detected TSS |
| 41 | 21.13% | Undetected TSS |
|  | | |
| **Distribution of the undetected TSS** | | |
| 41 | 100.00% | Undetected TSS |
| 19 | 46.34% | Signal too weak |
| 9 | 21.95% | Signal too weak, internal |
| 5 | 12.20% | Stepwise increase, no single position with strong signal |
| 8 | 19.51% | Stepwise increase, no single position with strong signal, internal |

**Table S2: Comparison of popular published read mapping visualization and analysis tools.** Here, we list the most important features of all compared tools. Features present in a tool are depicted by a "✓"and highlighted in green. Available features requiring a more detailed explanation are also highlighted in green. Features, which are present with constraints, are marked by "(✓)" and highlighted in yellow. Features, which are planned to be implemented, are shown as "🗶 (planned)" and highlighted in orange. All features not supported by a tool are marked by an "🗶" and highlighted in red.

| **Feature** | **Savant 2.0.4** | **IGB 8.0.1** | **Artemis 16.0** | **IGV 2.3.26 + Rockhopper 1.3.0** | **GenomeView 2450** | **ReadXplorer 1.9** |
| --- | --- | --- | --- | --- | --- | --- |
| Reference formats | BED, GFF3, GTF, Fasta | Genbank, GFF2/3, Fasta, BED | Genbank, EMBL, Fasta | BED, GFF2/3, GTF, Fasta | BED, EMBL, Genbank, GFF3, GTF, Fasta, PTT, TBL | Genbank, EMBL, GFF2/3, GTF, Fasta |
| Mapping formats | BAM | SAM, BAM | BAM, only one at a time or combination of multiple files | SAM, BAM | BAM | SAM, BAM, JOK |
| 6 frame view | 🗶 | 🗶 | ✓ | (✓) one strand at a time | ✓ | ✓ |
| Mapping classification | 🗶 | 🗶 | 🗶 | 🗶 | 🗶 | ✓ |
| Base/mapping quality shown | ✓ | ✓ | 🗶 | ✓ | ✓ | ✓ |
| Coverage graph | ✓ | ✓ | (✓) no position specific details | ✓ | ✓ | ✓ |
| Read pair visualization | ✓ | 🗶 | ✓ | ✓ | ✓ | ✓ |
| Alignment view | ✓ | ✓ | ✓ | ✓ | ✓ | ✓ |
| Histogram viewer | 🗶 | 🗶 | 🗶 | ✓ | ✓ | ✓ |
| Thumbnail viewer | 🗶 | 🗶 | 🗶 | 🗶 | 🗶 | ✓ |
| G/C plot | 🗶 | 🗶 | ✓ | 🗶 | 🗶 | 🗶 (planned) |
| Track comparison | 🗶 | 🗶 | 🗶 | 🗶 | ✓ | ✓ |
| Track combination | 🗶 | 🗶 | ✓ | ✓ | 🗶 | ✓ |
| Scaling | ✓ | ✓ | ✓ | ✓ | 🗶 | ✓ |
| Strand specific visualizations | (✓) only for coverage plot | ✓ | ✓ | (✓) only for alignments | ✓ | ✓ |
| Genetic code selection | 🗶 | 🗶 | 🗶 | ✓ | 🗶 | ✓ |
| SNP calling | ✓ | 🗶 | 🗶 | 🗶 | 🗶 | ✓ |
| VCF-support | ✓ | ✓ | ✓ | ✓ | ✓ | (🗶) ongoing |
| RNA-Seq analyses | Isoforms, abundancies, fragment length | 🗶 | Read count and RPKM calculation for selected features | Normalization, transcript assembly, RPKM, read count, operons | 🗶 | TSS, RPKM, read count, operon, coverage, feature coverage |
| Differential gene expression | egdeR | 🗶 | 🗶 | Similar to DESeq | 🗶 | DESeq, baySeq, Express Test |
| Continuous data support | TDF, BigWIG | BigWIG, BedGraph | 🗶 | TDF, BigWIG, WIG | TDF, BigWIG, WIG, pileup | 🗶 (planned) |
| Indexing | ✓ | 🗶 | ✓ except Fasta | ✓ except BAM | ✓ | ✓ |
| Bookmarks | ✓ | ✓ | ✓ | ✓ | 🗶 | 🗶 (planned) |
| Reformat data | Indexing and zipping BED, GenePred, GFF, GTF, VCF, WIG, BedGraph, BAM | 🗶 | Indexing BAM | Count, Sort, Index (except BAM), toTDF | Indexing  Fasta, BED,  BAM | Tab-separated (csv), Indexing BAM, SAM to BAM |
| Project based | ✓ | ✓ | ✓ | ✓ | ✓ | ✓ |
| Predefined genome list | ✓ | ✓ | 🗶 | ✓ | ✓ | 🗶 (planned) |
| Restriction sites | 🗶 | ✓ | 🗶 | 🗶 | 🗶 | 🗶 |
| Screenshots | ✓ | ✓ | ✓ | ✓ | ✓ | ✓ |
| Edit annotations | 🗶 | 🗶 | ✓ | 🗶 | ✓ | 🗶 (planned) |
| Pattern search | 🗶 | 🗶 | ✓ | ✓ | ✓ | ✓ |
| Database searches | 🗶 | NCBI | NCBI, pfam, rfam | 🗶 | NCBI | 🗶 (planned) |
| Export data | Fasta | BED, bedgraph | Fasta, PIR database, EMBL, Genbank, GFF, Sequin | BED, txt, TDM | EMBL,  GFF3 | xls |

Table S3: Analysis and import benchmark of ReadXplorer. This table shows the time and memory requirements for common tasks within the ReadXplorer software on a Windows 7 x64 Laptop with an i7-2630QM 2GHz processor and 8GB RAM. Additionally, the genome size and the number of mapped reads of the investigated data set is listed for each task. Reference imports include the genomic features and sequence. The "Overall Memory" column refers to the maximum observed memory usage during the task. The memory usage does not only include the memory footprint of the task, but the overall used memory for displaying the reference with genomic features, the data sets used in the task and the task itself. Further note that the observed memory footprint might be higher than necessary, since ReadXplorer was run with more than 1GB of available memory. This means, that the Java garbage collector does not always clean up the memory and possibly unneeded objects are still in memory.

| **Task** | **Genome Size** | **Mapped reads** | **Time** | **Overall Memory** |
| --- | --- | --- | --- | --- |
| Import/index *Pseudomonas aeruginosa* | ~ 6.26 mb | - | 8 s | 150 mb |
| Import/index *Caenorhabditis elegans* | ~ 99 mb | - | 14 s | 200 mb |
| Import/index *Homo sapiens* | ~ 30 gb | - | 8:05 min | 500 mb |
| Import *P. aeruginosa* reads | ~ 6.26 mb | 22,482,000 | 45:23 min | 900 mb |
| Import *P. aeruginosa* reads | ~ 6.26 mb | 16,000,000 | 30:25 min | 800 mb |
| Import *P. aeruginosa* reads | ~ 6.26 mb | 8,885,000 | 18:12 min | 800 mb |
| Import *Chlamydomonas reinhardtii* reads | ~ 120 mb | 82,000,000 | 151 min | 1000 mb |
| DESeq analysis | ~ 3 mb | 19,400,000 | 1:29 min | 510 mb |
| DESeq analysis | ~ 120 mb | 82,000,000 | 8:39 min | 800 mb |
| baySeq analysis | ~ 3 mb | 19,400,000 | 6:03 min | 390 mb |
| baySeq analysis | ~ 120 mb | 82,000,000 | 62 min | 800 mb |
| Express test | ~ 3 mb | 10,770,000 | 45 s | 400 mb |
| Express test | ~ 120 mb | 82,000,000 | 5:23 min | 700 mb |
| TSS detection | ~ 3 mb | 7,839,000 | 19 s | 510 mb |
| TSS detection | ~ 3 mb | 17,069,000 | 1:10 min | 440 mb |
| TSS detection | ~ 120 mb | 82,000,000 | 6:27 min | 480 mb |
| RPKM and read count calculation | ~ 3 mb | 7,839,000 | 25 s | 520 mb |
| RPKM and read count calculation | ~ 120 mb | 82,000,000 | 2:32 min | 860 mb |
| Operon detection | ~ 3 mb | 17,069,000 | 36 s | 430 mb |
| Operon detection | ~ 120 mb | 82,000,000 | 5:19 min | 590 mb |
| Coverage analysis | ~ 6.26 mb | 12,115,000 | 37 s | 420 mb |
| Coverage analysis | ~ 120 mb | 82,000,000 | 3:19 min | 480 mb |
| Feature Coverage analysis | ~ 6.26 mb | 12,115,000 | 41 s | 480 mb |
| Feature Coverage analysis | ~ 120 mb | 82,000,000 | 3:20 min | 560 mb |
| SNP detection (55,000 SNPs found) | ~ 6.26 mb | 12,115,000 | 7:03 min | 480 mb |
| SNP detection (57.000 SNPs found) | ~ 120 mb | 82,000,000 | 8:35 min | 710 mb |

**Figure S11: Simulated data set evaluation.** Comparison of a mapped *E. Coli* K-12 MG1655 read data set simulated by wgsim (<https://github.com/lh3/wgsim>) with two conditions (normal and down regulation of 100 selected genes), each of them having 8 replicates. The down regulated genes are randomly assigned to 20 bins. Each bin simulates a different intensity of down regulation and contains five genes. Starting with the depletion of 5% of the reads for the genes in the first bin, the down regulation is increased by 5% in each bin, resulting in a down regulation of 100% (complete knock out without reads) for the five genes in the last bin. The default options and no "start offset" were chosen for the subsequent analysis of the data set with all three tools from within ReadXplorer. Results were sorted by best statistical confidence as given by baySeq (FDR) and DESeq (padj) and fold change. The Express Test is a method to get rapid insight into the data and does not provide a significance level, thus the result has been sorted by fold change only. Ultimately, we compared if the 100 stepwise down regulated genes are detected as differentially expressed and how well they are assigned to their corresponding bins, i.e. the most strongly down regulated genes are expected to be found at the top of the result list after sorting. The figure shows the result of the assignment into the 20 predefined bins (left) and the number of genes of the 100 down regulated genes, which have been detected by each tool (right). baySeq and Express Test assign most genes to the correct bin, while DESeq places them in neighboring bins more often (left).

In the histogram on the right, only genes which are among the top 100 results after sorting are counted. As DESeq and baySeq provide a significance level, the counting has been restricted to genes with a padj or FDR below 0.05. Only 4-6 genes are not present in the top 100 differentially expressed genes of all three tools (right). In total DESeq's padj is more conservative than baySeq's FDR: DESeq detects 96 genes as differentially expressed of which 2 are not in the list of the 100 down regulated genes, whereas baySeq detects 107 genes, of which 14 are not in the list of the 100 down regulated genes (dark grey), with an FDR below 0.05. The Express Test shows the best overall performance in this analysis, but it is important to note, that the Express Test does not perform a statistical test for differential gene expression like the other two tools. Its confidence value is rather meant to constitute the variation within the replicates.
